# Herbal medicine Yinchenhaotang protects against α-naphthylisothiocyanate-induced cholestasis in rats

**DOI:** 10.1038/s41598-017-04536-5

**Published:** 2017-06-23

**Authors:** Jingyu Yan, Guoxiang Xie, Chungeng Liang, Yiyang Hu, Aihua Zhao, Fengjie Huang, Ping Hu, Ping Liu, Wei Jia, Xiaoning Wang

**Affiliations:** 1 E-institute of Shanghai Municipal Education Commission, Shanghai University of Traditional Chinese Medicine, Shanghai, 201203 China; 2 Institute of Liver Disease, Shuguang Hospital, Shanghai University of Traditional Chinese Medicine, Shanghai, 201203 China; 3 University of Hawaii Cancer Center, Honolulu, Hawaii 96813 USA; 4 Center for Translational Medicine, Shanghai Jiao Tong University Affiliated Sixth People’s Hospital, Shanghai, 200233 China; 5 Laboratory of Functional Materials Chemistry, School of Chemistry and Molecular Engineering, East China University of Science and Technology, Shanghai, 200237 China

## Abstract

Cholestasis is a clinical disorder defined as an impairment of bile flow, and that leads to toxic bile acid (BA) accumulation in hepatocytes. Here, we investigated the hepatoprotective effect of Yinchenhaotang (YCHT), a well-known formulae for the treatment of jaundice and liver disorders, against the cholestasis using the α-naphthylisothiocyanate (ANIT)-induced cholestasis in male Wistar rats. ANIT feeding induced significant cholestasis with substantially increased intrahepatic retention of hydrophobic BAs. The dynamic changes of serum and liver BAs indicated that YCHT was able to attenuate ANIT-induced BA perturbation, which is consistent with the histopathological findings that YCHT significantly decreased the liver damage. YCHT treatment substantially reduced serum alanine aminotransferase (ALT), alkaline phosphatase (AST), total bilirubin (TBIL) and direct bilirubin (DBIL) with minimal bile duct damage in the ANIT treated rats. Elevated mRNA expression of liver IL-6, IL-17A, IL-17F, TGF-β1, α-SMA, TGR5, NTCP, OATP1a1, and ileum ASBT and decreased liver IL-10, FXR, CAR, VDR, BSEP, MRP2, MRP3, MRP4 was also observed in ANIT-induced cholestasis but were attenuated or normalized by YCHT. Our results demonstrated that the BA profiles were significantly altered with ANIT intervention and YCHT possesses the hepatoprotective potential against cholestatic liver injury induced by hepatotoxin such as ANIT.

## Introduction

Cholestasis, an impairment in bile formation, is a common feature in many human liver diseases^1^. Bile acids (BAs) are metabolites synthesized from cholesterol in the liver that are normally secreted into bile^2^, gaining increasing recognition as important metabolic signaling molecules that modulate lipid, glucose, and energy metabolism^3^. Failure of biliary BA excretion in cholestasis leads to retention and accumulation of hydrophobic BAs within the liver^4^. The accumulation of potentially toxic BAs results in hepatocellular damage followed by inflammation, fibrosis and liver cirrhosis^5^. The accumulation of BA inside hepatocytes is the major cause of cholestatic liver damage^6^, including structural and functional injuries of hepatocyte membranes^7^, cell death^8^ and activation of inflammatory and fibrogenic signaling pathways^9^. Accumulated BA within hepatocytes impairs mitochondrial respiration and electron transport and stimulates the generation of reactive oxygen species (ROS) in hepatic mitochondria^10^. Several studies have suggested an important role of increased oxidative stress in the pathogenesis of cholestatic injury^11, 12^. Accordingly, mitochondrial free radicals may then modify nucleic acids, proteins, and lipids. In fact, an increase in lipid peroxidative products has been observed in cholestatic livers^13^. Besides the accumulation of BAs in hepatocytes and circulation, the elevated plasma levels of bilirubin are also a laboratory marker of cholestasis. Although the fact that those high levels of BA in hepatocytes and circulation are known, however, there are very few reports that systemically delineate the composition of BA profiles in the liver and blood pool, and determine how different BAs and different BA concentrations can affect hepatocytes during cholestasis.

**Figure 1:**
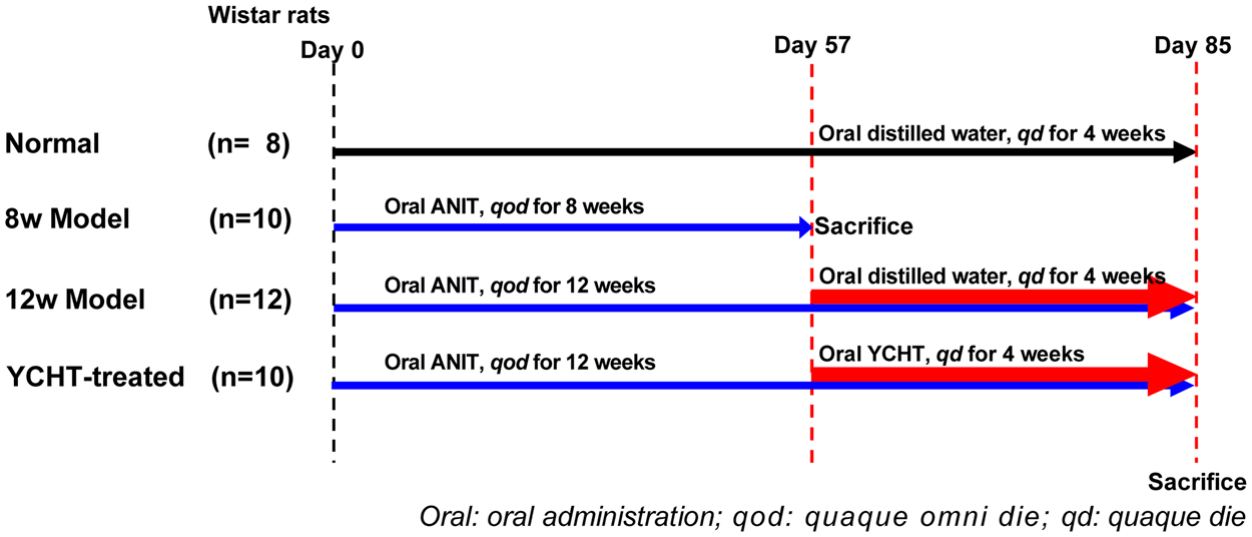
Scheme of the experiment.

Yinchenhaotang (YCHT, Inchin-ko-to or TJ-135 in Japan) is one of the most important and widely used traditional Chinese medicine in clinical practice for jaundice and liver disorders treatment^14^, which is composed of *Artemisia annua* L., *Gardenia jasminoides* Ellis, and *Rheum Palmatum* L. It was originally recorded by ‘Shanghanlun’ in the Han Dynasty of China (150–215A.D.) and was officially listed in the Chinese Pharmacopoeia^15^. It has been demonstrated to be able to attenuate alpha-naphthylisothiocyanate (ANIT)^16^ or other toxions^17, 18^-induced intrahepatic cholestasis in experimental animals. A variety of biological effects of YCHT has been described, such as treat cholestasis, liver fibrosis, hepatitis C, biliary cirrhosis, and cholestatic liver diseases^19, 20^. Since YCHT has a hepatoprotective property against liver injury induced by various toxicants, it appears to be an attractive formula for therapy of cholestatic liver diseases. Although such herbal formula has distinct pharmacological effects, its hepatoprotective mechanisms of action remain unclear. To determine the protective effect of YCHT on the intrahepatic cholestasis, we used ANIT as the model of intrahepatic cholestasis in rats. The animal study was performed as shown in Fig. 1. The results obtained from this study will provide a novel therapeutic option of YCHT for intrahepatic cholestasis diseases.

Here, we analyzed the BA profiles of the serum and liver in ANIT-induced cholestasis male Wistar rats using ultra-performance liquid chromatography triple quadrupole mass spectrometry (UPLC-TQMS). We measured the expression of different genes involved in inflammation and BA metabolism. We also used YCHT treatment in the same animal model to gain mechanistic insights into their protective effects against ANIT-induced cholestasis.

## Results

### Active composition in the YCHT extract

There are four main active compounds, gallic acid, chlorogenic acid, geniposide and crocin in the YCHT extraction and their levels were measured using HPLC (Supplementary Fig. S1). The contents of gallic acid are 4.54 mg/g, chlorogenic acid are 42.35 mg/g, geniposide are 77.59 mg/g and crocin are 1.85 mg/g. YCHT has been safely used with few adverse events in clinic^21^. It has been reported that geniposide in *Gardenia jasminoides* Ellis showed liver toxicity when rats were given geniposide at a dose of 228 mg/kg body weight^22^. However, in our study, the concentration of geniposide administrated was 120 mg/kg, which is much lower than the dosage that will induce liver toxicity.

### General information of the ANIT-induced cholestasis

As shown in Table 1, the body weight of rats in ANIT group was significantly decreased compared to the normal, while in YCHT treated group, the body weight was slightly increased compared to the rats in ANIT group at week 8.

**Table 1:** Biochemical Parameters of Serum and Liver^*a*^.

Parameters	Normal	8w Model	12w Model	YCHT-treated
n	8	10	12	10
Body weight (g, mean ± SD)	474.5 ± 57.24	349.3 ± 25.13**	396.2 ± 32.46**##	380.7 ± 19.70
Liver weight (g, mean ± SD)	12.65 ± 2.27	13.56 ± 1.39	13.91 ± 1.26	14.43 ± 0.96
Liver/body weight ratio (%)	2.6 ± 0.2	3.8 ± 0.2**	3.5 ± 0.1**#	3.8 ± 0.2†
ALT (IU/L)	71.38 ± 19.99	137.6 ± 85.49	205.6 ± 81.91**#	78.86 ± 23.25††
AST (IU/L)	144.8 ± 24.74	181.7 ± 143.2	266.5 ± 88.6*#	141.7 ± 35.4†
ALB (g/L)	30.05 ± 4.6	26.12 ± 2.74*	28.54 ± 4.34*	31.19 ± 3.17†
ALP (IU/L)	85.38 ± 16.40	154.30 ± 49.18**	136.90 ± 33.04**	118.40 ± 39.54†
TBIL (μmol/L)	1.60 ± 0.44	4.21 ± 2.62*	6.92 ± 3.11**##	3.00 ± 1.19††
DBIL (μmol/L)	0.80 ± 0.39	3.27 ± 2.35**	5.49 ± 2.80**##	2.11 ± 0.83††
TG (mmol/L)	0.98 ± 0.33	0.47 ± 0.06**	0.58 ± 0.08**	0.47 ± 0.12
TC (mmol/L)	2.41 ± 0.57	2.51 ± 0.36	2.84 ± 0.27**	2.44 ± 0.37†
Cr (μmol/L)	42.38 ± 9.07	42.50 ± 5.64	46.91 ± 8.71	41.00 ± 7.77
BUN (mmol/L)	5.73 ± 0.85	4.13 ± 0.76**	5.67 ± 0.63##	5.94 ± 1.66
γ-GT (U/L)	9.84 ± 3.407	30.75 ± 4.06**	46.37 ± 5.518**#	32.16 ± 1.697††
HYP (μg/g liver tissue)	234.5 ± 13.1	460.2 ± 33.21**	641.7 ± 77.81**##	470.4 ± 36.68†

^a^Significant differences were determined by One-Way ANOVA. Asterisk (*) indicates *P* < 0.05, **P < 0.01 *vs*. normal group. ^#^ *P* < 0.05, ^##^ *P* < 0.01, *vs*. 8w model group. Dagger (†) indicates *P* < 0.05, ^††^ *P* < 0.01, *vs*. 12w model group. Data are presented as mean ± SEM.

The serum levels of ALT, AST, alkaline phosphatase (ALP), TBIL, DBIL, total cholesterol (TC) and liver hydroxyproline (HYP) were markedly increased at week 8 and 12 due to ANIT intervention (p < 0.05, Table 1) but subsequently decreased after YCHT treatment. The levels of triglyceride (TG) were significantly decreased in rats at week 8 and 12 due to ANIT intervention (p < 0.05, Table 1) and subsequently increased after YCHT treatment. Serum levels of ALT, AST, albumin (ALB), TBIL, DBIL, and blood urea nitrogen (BUN), liver γ-glutamyl transpeptidase (γ-GT) activity and HYP content in liver tissue were higher at week 12 than those at week 8, suggesting the gradually aggravated cholestatic liver injury.

### Histopathological evaluation

**Figure 2:**
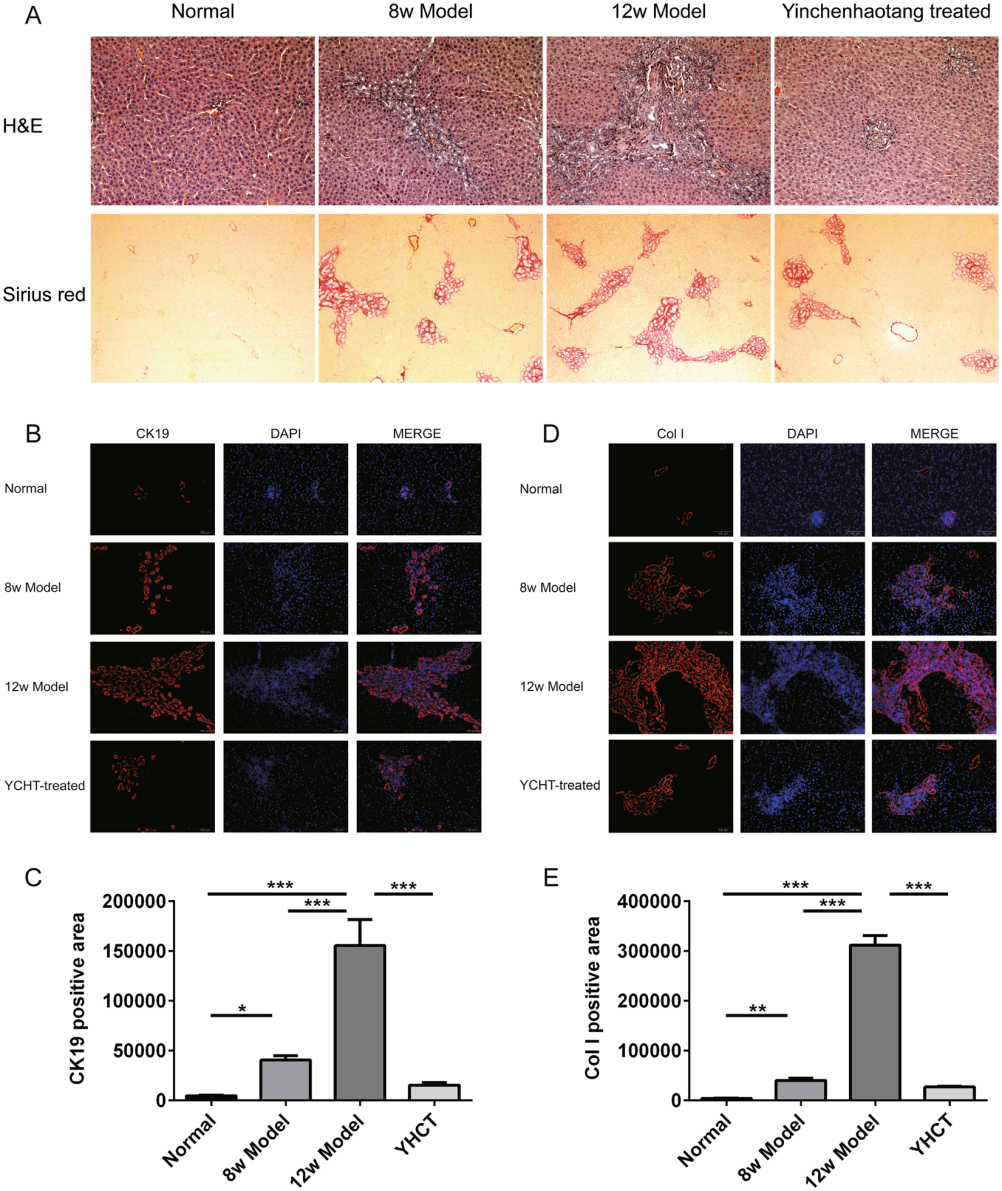
Histological features of ANIT-induced cholestasis model. (**A**) H&E and Sirius red stained liver sections from normal group, 8w model group, 12w model group and YCHT-treated group. Normal, healthy rats; 8w Model, ANIT treatment for 8 weeks; 12w Model, ANIT treatment for 12 weeks; YCHT-treated, ANIT-induced cholestasis rats treated with YCHT from 9^th^ week to 12^th^ week. Original magnifications × 100 for H&E, and × 200 for Sirius red. (**B**) Immunofluorescence analysis of CK19 in liver sections. (**C**) The positive area of CK19 in different groups. (**D**) Immunofluorescence analysis of Collagen I in liver sections. (**E**) The positive area of Collagen I in different groups. *P < 0.05; **P < 0.01; ***P < 0.001.

H&E staining of liver tissue showed that all rats in normal group appeared normal. At week 8, obvious biliary epithelial cell proliferation were occurred in the portal area, formatting a large number of irregularly arranged small bile duct and inflammatory cell infiltration. With the prolonged action time of ANIT, the liver presented large focal necrosis, island structure of various sizes, obvious biliary epithelial cell proliferation, a large number of irregularly arranged small bile duct and inflammatory cell infiltration with significantly reduced hepatocytes (Fig. 2A). Sirius red staining demonstrated that the original lobule was altered with a large number of collagen deposition around proliferated small bile ducts (Fig. 2A). Compared with the model group, YCHT treatment ameliorates the ANIT induced biliary epithelial cell proliferation and the degree of liver fibrosis. Liver samples were also harvested for immunofluorescence analysis of cytokeratin-19 (CK19) and Collagen I (Fig. 2B and C). Cytokeratins are the major structural proteins of intermediate filaments in epithelial cells; CK19 is an antigenic marker for bile duct epithelial cells and bile ductules^23^. CK19-positive cells (red fluorescence) were expressed only in the portal area in normal liver tissue. With the increased time of ANIT intervention, CK19-positive cells in the portal area increased gradually, suggesting that ANIT intervention significantly increased bile duct epithelial cell proliferation. Collagen I was found to be widely distributed in the liver tissue of ANIT model rats compared with the normal rats. The expression of Collagen I increased gradually with the prolonged time of ANIT intervention. YCHT intervention significantly reduced the expression of CK19 and Collagen I in hepatic tissue of model rats (Fig. 2D and E).

### Chronic ANIT intervention alters the expression of pro-inflammatory cytokines and the effect of YCHT

**Figure 3:**
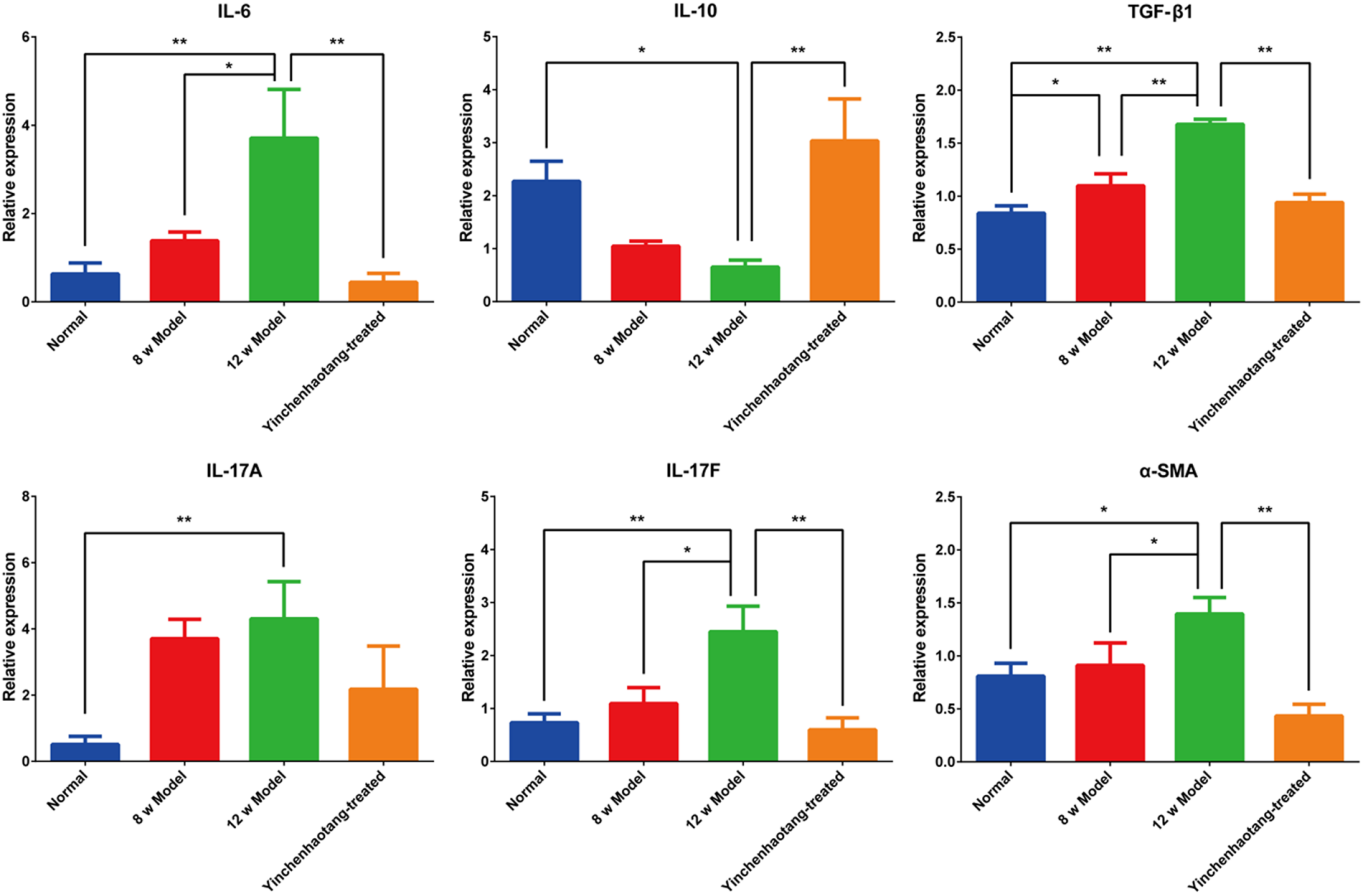
mRNA expression of IL-6, IL-10, TGF-β1, IL-17A, IL-17F, and α-SMA in the liver. Normal, healthy rats; 8w Model, ANIT treatment for 8 weeks; 12w Model, ANIT treatment for 12 weeks; Yinchenhaotang-treated, ANIT-induced cholestasis rats treated with Yinchenhaotang from 9^th^ week to 12^th^ week. All data are presented as mean ± SEM. (n = 8–12; *P < 0.05; **P < 0.01).

Inflammation is associated with the ANIT-induced cholestasis and hepatocellular injury. As compared with the normal rats, ANIT intervention significantly increased the mRNA expression of transforming growth factor β1 (TGF-β1), interleukin-6 (IL-6), interleukin-17F (IL-17F), interleukin-17A (IL-17A), and α-smooth muscle actin (α-SMA) and significantly decreased the mRNA expression of interleukin-10 (IL-10) (*P* < 0.05). They were attenuated or normalized after YCHT treatment (Fig. 3).

### Serum and liver BA alterations in ANIT-induced cholestatic rats and the effect of YCHT treatment

**Figure 4:**
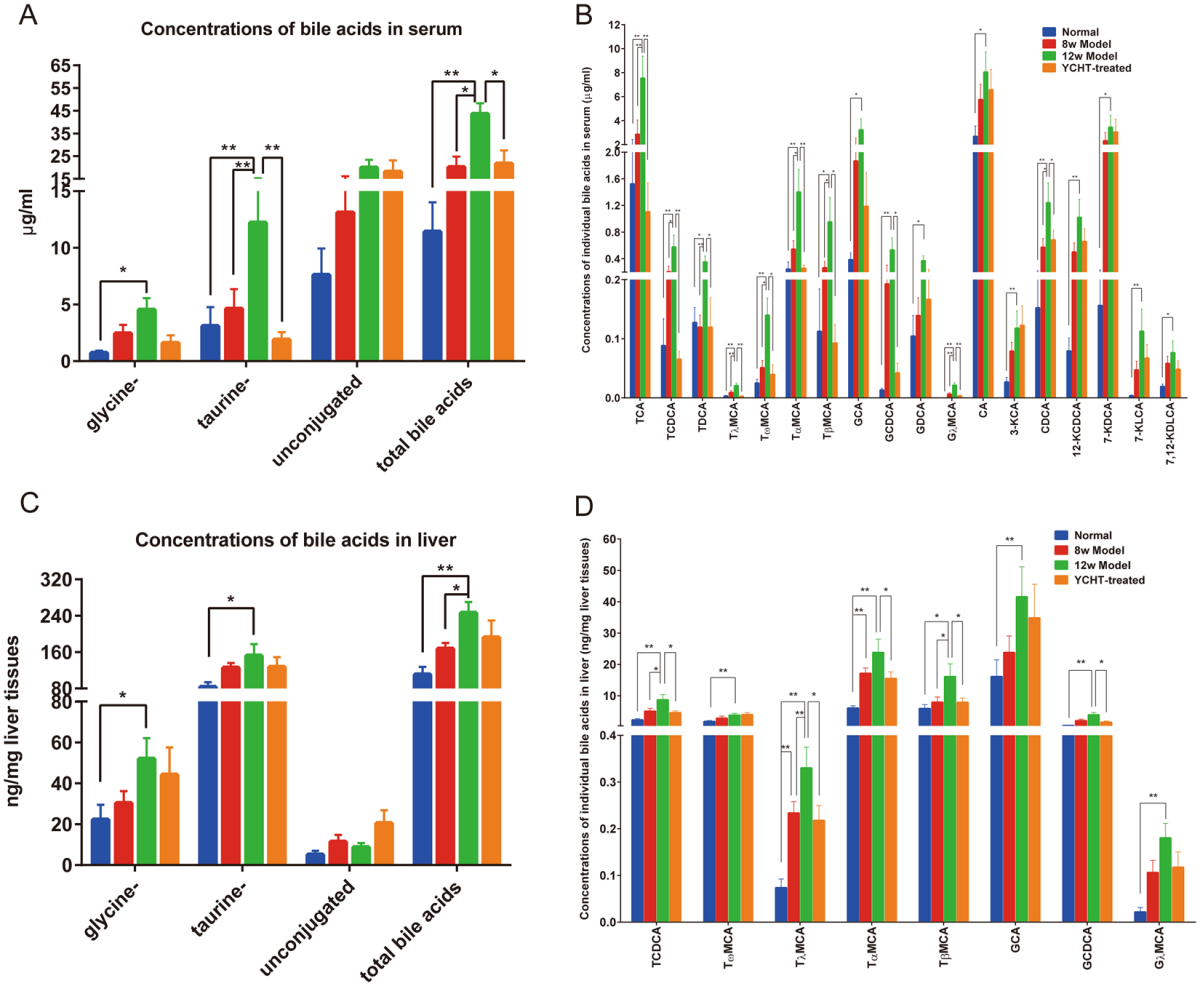
ANIT induced significant alteration of BA profile in serum and liver (mean ± SEM). (**A**) Concentrations of unconjugated, taurine-conjugated, glycine-conjugated BAs and total BAs in serum, (**B**) Concentrations of individual BAs in serum, (**C**) Concentrations of unconjugated, taurine-conjugated, glycine-conjugated BAs and total BAs in the liver, and (**D**) Concentrations of individual BAs in the liver. Data are presented as mean ± SEM of 5–9 rats. *P < 0.05, **P < 0.01.

The serum BA profile was different from that of the liver; this is potentially because the BA concentrations measured in tissues predominantly derived from tissue BA and not from residual blood contained within the tissue^24^. In serum from control rats, unconjugated BAs were most abundant. The levels of both unconjugated and glycine- and taurine-conjugated BAs were increased in the serum following ANIT intervention but decreased after YCHT treatment (Supplementary Table S1 and Fig. 4A). Specially, the levels of taurochenodeoxycholate (TCDCA), tauro α-muricholate (Tα-MCA), tauro β-muricholate (Tβ-MCA), tauro λ-muricholate (Tλ-MCA), glycochenodeoxycholate (GCDCA), taurocholate (TCA), taurodeoxycholate (TDCA), tauro ω-muricholate (Tω-MCA), glycol λ-muricholate (Gλ-MCA) and chenodeoxycholate (CDCA) were significantly increased due to ANIT intervention but were normalized after YCHT treatment (Fig. 4B).

In the livers of rats, taurine-conjugated BAs were predominated; however, ANIT intervention resulted in a significant increase in both glycine-conjugated and taurine-conjugated BAs (Supplementary Table S2 and Fig. 4C). Additionally, the levels of TCDCA, GCDCA, Tα-MCA, Tβ-MCA, and Tλ-MCA were increased in ANIT-treated rats compared to the controls but decreased after YCHT treatment (Fig. 4D). Glycine- and taurine-conjugated BAs were significantly elevated in the liver following ANIT intervention and decreased after YCHT treatment; similar to what was observed in serum samples (Fig. 4).

### YCHT regulated gene expression involved in bile acid metabolism

**Figure 5:**
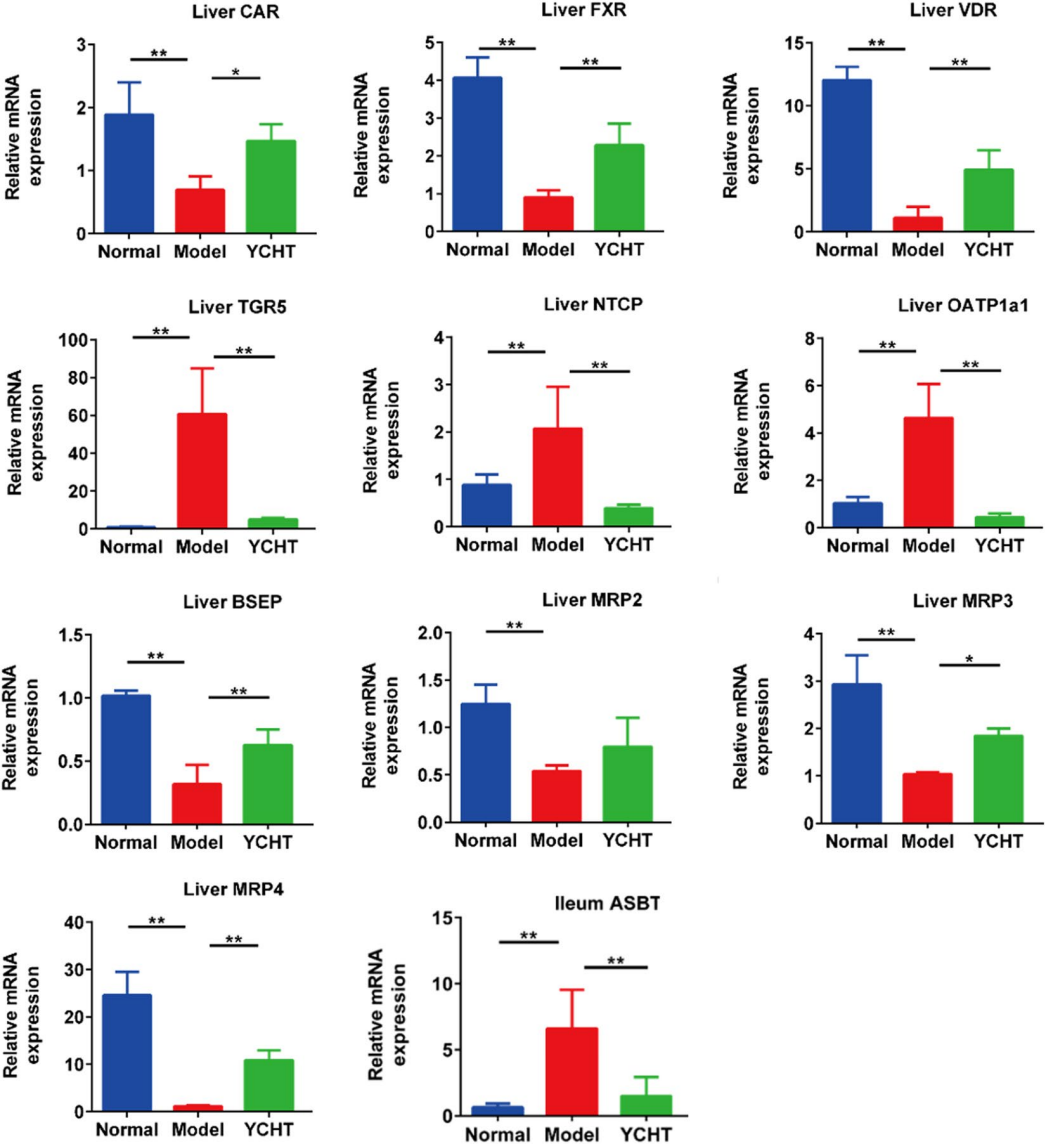
The mRNA expression of liver FXR, CAR, VDR, TGR5, NTCP, BSEP, OATP1a1, MRP2, MRP3, and MRP4, and ileum ASBT. Normal, healthy rats; Model, ANIT treatment for 12 weeks; YCHT, ANIT-induced cholestasis rats treated with Yinchenhaotang from 9^th^ week to 12^th^ week. All data are presented as mean ± SEM. (n = 8–12; *P < 0.05; **P < 0.01).

To further clarify the effective mechanisms of YCHT, the relative expressions of key genes involved in bile acid metabolism were measured. As shown in Fig. 5, the levels of liver farnesoid X receptor (FXR), constitutive androstane receptor (CAR), and vitamin D receptor (VDR) mRNAs were decreased (*P* < 0.01), and G protein–coupled bile acid receptor (TGR5) were increased (*P* < 0.01) in ANIT group, and all of them restored to normal levels by YCHT treatment. Furthermore, the levels of mRNAs related BA absorption such as organic anion-transporting polypeptides (OATP1) and Na^+^-taurocholate cotransport peptide (NTCP) were significantly up-regulated in ANIT group (*P* < 0.01), and related excretion such as multidrug-resistance associated protein (MRP3), MRP2, MRP4 and bile salt export pump (BSEP) were significantly down-regulated (*P* < 0.01). ANIT also significantly increased the expression of ileum apical sodium-dependent bile acid transporters (ASBT).

**Figure 6:**
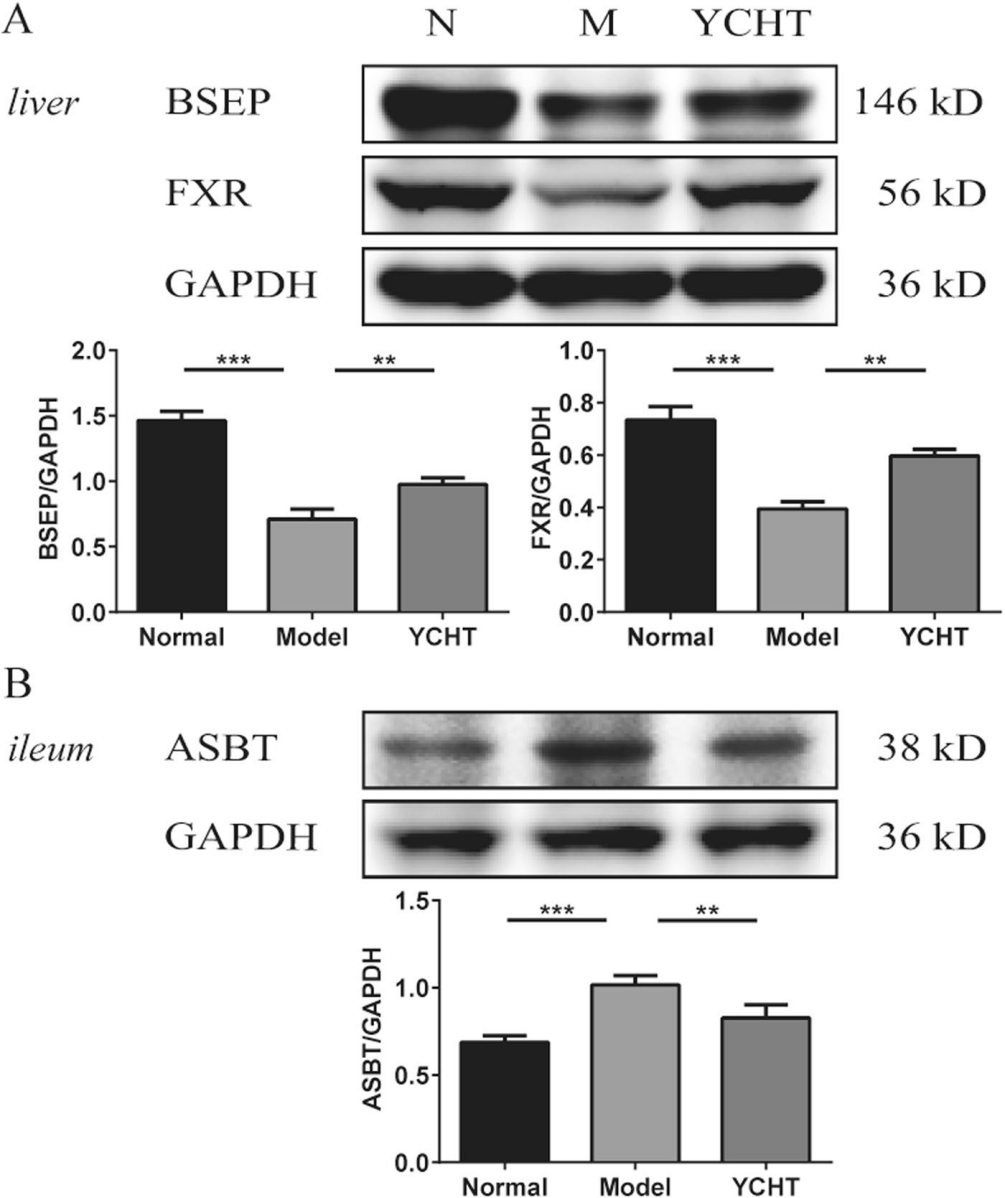
The protein expression of liver FXR, BSEP, and ileum ASBT. (**A**) The protein levels of FXR and BSEP in liver tissue were significantly decreased and (**B**) the protein levels of ASBT in ileum were significantly increased after ANIT-induced cholestasis model but were normalized after YCHT intervention. **P < 0.01; ***P < 0.001. The full-length gels and blots are provided in the Supplementary Information.

The mRNA expression of FXR, BSEP and ASBT were also validated by Western blot. The protein levels of FXR and BSEP were significantly decreased while ileum ASBT levels were significantly increased in ANIT model rats, but they were all normalized after YCHT intervention (Fig. 6 and Supplementary Fig. S2).

### Correlation between BAs and bilirubin

**Figure 7:**
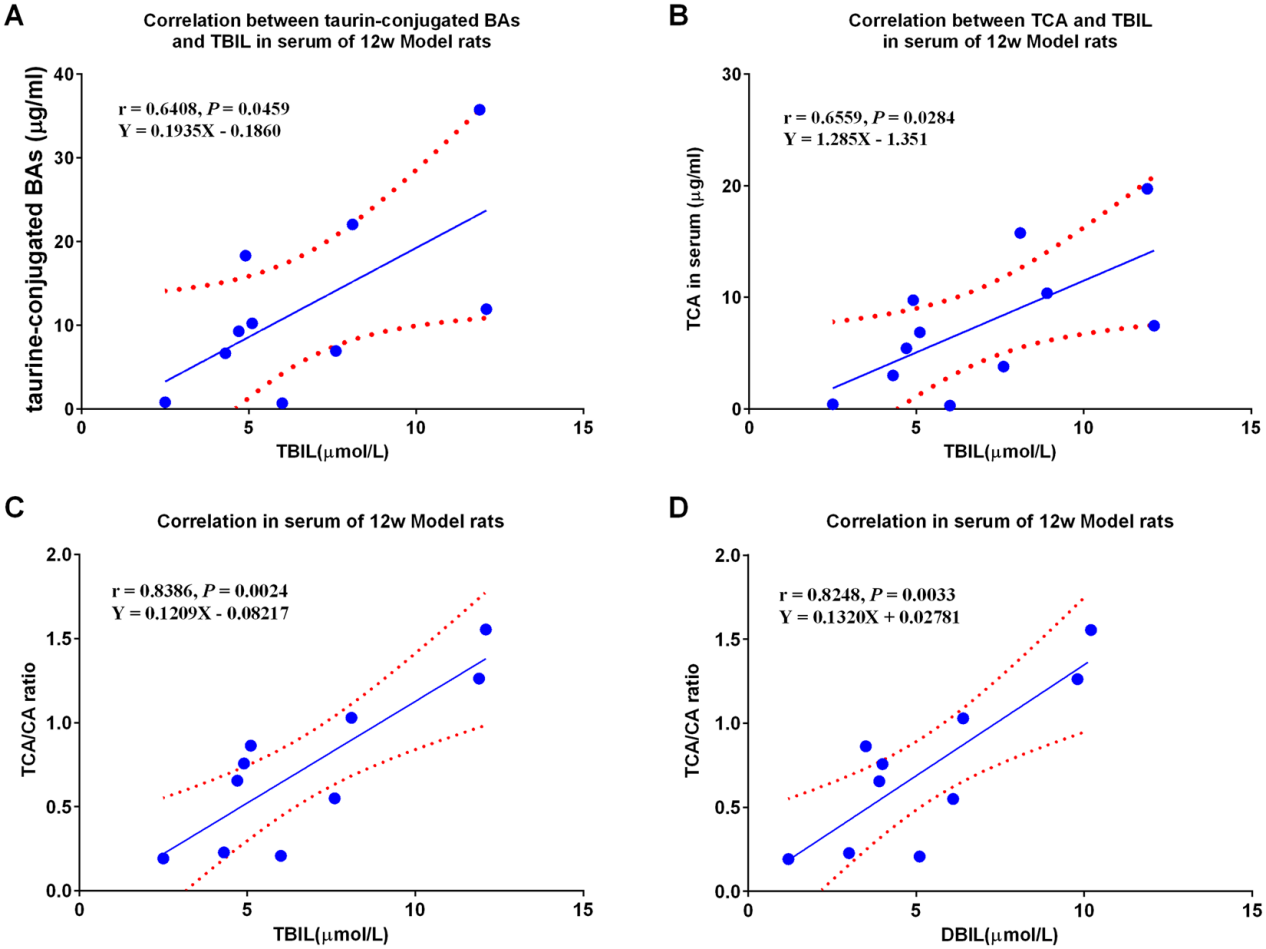
Correlation analysis between serum BAs and bilirubin. (**A**) taurine-conjugated BAs *vs*. TBIL; (**B**) TCA *vs*. TBIL; (**C**) ratio of TCA to CA *vs*. TBIL; and (**D**) TCA/CA *vs*. DBIL.

Pearson correlation analysis showed no correlation between serum BAs and bilirubin in model rats. A low correlation exists between serum BAs and bilirubin in model rats at week 8, and at week 12, a significant correlation was observed between serum BAs and bilirubin. The serum taurine-conjugated BAs was significantly positively correlated with TBIL (r = 0.6408, *P* = 0.0459), among which, TCA was the most significant (r = 0.6559, *P* = 0.0284). Interestingly, the ratio of TCA to CA was significantly positively correlated with TBIL (r = 0.8386, *P* = 0.0024) and DBIL (r = 0.8248, *P* = 0.0033) (Fig. 7).

## Discussion

ANIT is a widely used chemical to mimic human intrahepatic cholestasis. ANIT is conjugated with glutathione in hepatocytes and transported through the canalicular efflux transporter (MRP2) into bile, where ANIT dissociates from glutathione. The released ANIT selectively damages bile duct epithelial cells and causes cholangitis and subsequent intrahepatic cholestasis^25^. In response to ANIT, bile duct epithelial cells release pro-inflammatory cytokines, such as tumor necrosis factor-α (TNF-α) and monocyte chemoattractant protein-1 (MCP-1). Increases in inflammatory cytokines result in migration and infiltration of neutrophils, leading to lipid peroxidation and bile duct epithelial cell damage^26–28^. Early studies into the mechanisms of cholestatic liver injury strongly implicated BA- induced apoptosis as the major cause of hepatocellular injury. Recent work has focused both on the role of BAs in cell signaling as well as the role of sterile inflammation in the pathophysiology. Advances in modern analytical methodology have allowed for more accurate measuring of BA concentrations in serum and liver to very low levels of detection. This study showed both how different BAs and different BA concentrations can affect hepatocytes during cholestasis, and additionally provide insight into how these data support recent hypotheses that cholestatic liver injury may not occur through direct BA-induced apoptosis, but may involve largely inflammatory cell-mediated liver cell necrosis.

In this study, we examined the BA profiles of the liver and serum in normal rats and determined how those profiles were impacted by ANIT intervention. Serum ALT and AST, the widely used markers in evaluating the degree of liver injury^29^, and bilirubin including DBIL and TBIL, were markedly increased due to ANIT intervention and then significantly decreased with YCHT treatment. These results are in line with earlier reports showing that YCHT reduces increases in ALT, AST, DBIL and TBIL levels in ANIT-induced cholestasis model^17, 30^. It has also been reported that YHCT can alleviate obstructive biliary cirrhosis induced by bile duct ligation in rats via inhibiting biliary epithelial cell proliferation and activation^31^. Our previous study also demonstrated that YHCT administration alleviated liver fibrosis via inhibiting hepatic stellate cell (HSC) activation in dimethylnitrosamine-treated rats^32, 33^. Studies by Takahashi *et al*. showed that YHCT significantly mitigated the necroinflammation in the high fat diet induced NASH mice^34^.

Chronic ANIT intervention resulted in a global alteration of BA profiles in the liver and blood. Elevated hepatic tissue BA concentrations induce hepatocellular injury by causing hepatocyte apoptosis^35–37^. Retention of bile constituents within the hepatocyte during cholestasis is associated with hepatocyte apoptosis^38^. Although the mechanisms of cholestasis associated with hepatocyte apoptosis are likely complex and multifactorial, hydrophobic BAs are especially hepatotoxic and accumulated in the liver in cholestatic disorders^39^. The failure to secrete BAs into bile results in liver injury, cirrhosis, and death from liver failure^40^. The BA profiles for liver tissues in normal and the ANIT-treated rats were dominated by taurine- and glycine-conjugated BAs, with unconjugated BAs present in small amounts. Generally, ANIT intervention led to a substantial increase in glycine- and taurine-conjugated BAs in liver tissue examined, even during short-term ANIT intervention (8 weeks). In contrast, unconjugated BAs were predominant in the serum and all BAs were increased under the same conditions, and the alterations in the BA profile in the blood were normalized after YCHT treatment. Taurine-conjugated BAs are more hydrophilic and less toxic than either unconjugated or glycine-conjugated BAs. Liver BAs in normal rats were predominantly taurine conjugated, consistent with the findings of Dupont *et al*.^41^, who saw that one-half of the liver BAs in miniature swine were conjugated with taurine. Since YCHT intervention can have a profound impact on the gut microbiota^42, 43^, it is possible that the reduced abundance of taurine-conjugated BAs in YCHT-treated rats is at least partially due to an YCHT-induced disturbance of the gut microbiota.

FXR plays a central role in the regulation of BA synthesis, excretion, and transport^44, 45^. Activation of FXR inhibits BA synthesis from cholesterol and activates secretion into bile canaliculi through BSEP, therefore protecting against the toxic accumulation of BAs in hepatocytes^46^. We observed that the FXR and BSEP expression in rat liver was significantly decreased in the ANIT model, suggesting a mechanism of down-regulating hepatic efflux transporters, thus leading to increased BA accumulation in hepatocytes and BA-induced liver injury. The higher levels of BAs in ANIT-induced model rats were also probably due to bile duct obstruction in liver impairments and impaired BAs enterohepatic circulation, with reduced transformation of BA to intestine and the feedback from intestine^47^. Although the exact mechanisms underlying the increase of FXR expression in YCHT-treated rats are unclear, we propose that YCHT-induced alterations in the gut microbiota may play a critical role in this process. In the terminal ileum and colon, BAs are reabsorbed by the enterocytes via ASBT and released into the portal vein for return to the liver. The significantly increased ASBT also led to increased BA accumulation in hepatocytes^48^. BAs can be reabsorbed into hepatocytes through both NTCP and OATP^49^. The levels of both NTCP and OATP1a1 mRNAs, were found increased in ANIT- treated rats, which may lead to increased BA levels in hepatocytes.

Gene expression analysis indicated that ANIT intervention led to alteration of genes involved in inflammation. Inflammation is a critical component of cholestasis. Abnormal expression of IL-6, IL-10, IL-17A, IL-17F, TGF-β1 and α-SMA are closely associated with the cholestasis. IL-6 is an important mediator of inflammation *in vivo*. The hepatocytes, Kupffer cells, and HSC secrete large quantities of IL-6 in the liver disease state. High concentrations of IL-6 can stimulate hepatocyte apoptosis and HSC proliferation, activate Kupffer cells to secrete more pro-inflammatory cytokines, and promote extracellular matrix synthesis, leading to aggravated liver fibrosis. IL-6 can directly activate signal transducers and activators of transcription (STAT3), increase the expression of RORγt and induce naive T cells to CD4 + Th17 cells differentiation^50^. Also, mice CD4 + CD25-Foxp3-T cells and CD4 + CD25 + Foxp3 + Treg cells can be transformed into Th17 cells in the presence of IL-6 without the addition of exogenous TGF-β1, resulting in reduced number of Treg and increased number of Th17^51^. In the progression of liver fibrosis, activated Kupffer and HSC cells secrete large amounts of IL-6 and TGF-β1, promote the transformation from Treg to Th17, resulting in reduced secretion of IL-10 and increased secretion of IL-17. IL-10 is an important immunosuppresive cytokine and can effectively prevent inflammation and excessive immune responses. Various types of cells can produce IL-10^52^. IL-10 can inhibit Kupffer cells to produce inflammatory cytokine, tumor necrosis factor-α (TNF-α), and reduce the activation of HSC^53^. Clinical studies have demonstrated that long-term IL-10 therapy can reduce inflammation and fibrosis score of patients with hepatitis C virus^54^. In contrast, IL-10 knockout mice showed a more significant liver fibrosis than wild-type mice^55^. Previous reports indicated that serum IL-6 was highly expressed in cholestasis, which will be attenuated when the expression of IL-6 was reduced^56^. Serum IL 10-level was negatively correlated with TBIL concentrations in chronic liver injury^57^. It was reported that serum IL-17 was highly expressed in cholestatic patients and its level decreased after immunosuppressive therapy with improved cholestasis^58^. As a result, the up-regulated IL-10 expression and down-regulated of IL-6, IL-17A, IL-17F, TGF-β1 and α-SMA in YCHT may be indicative of the reduced levels of inflammation.

It was reported that serum BA and bilirubin concentrations changed in parallel throughout cholestatic viral hepatitis, chronic active hepatitis and alcoholic hepatitis^59^, which is accordance with our findings that the serum BAs and bilirubin were significantly positively correlated.

In conclusion, ANIT intervention had a profound effect on the BA profiles in the liver and serum. The ANIT-modulated BA profiles were characterized by a marked increase in unconjugated and taurine- and glycine-conjugated BAs. Gene expression analysis revealed several inflammation related cytokines were significantly altered including increased IL-6, IL-17A, IL-17F, TGF-β1 and α-SMA and decreased IL-10 expression. YCHT can attenuate the metabolic perturbation in ANIT-induced model rats, which was supported by liver tissue histology, and liver inflammation cytokine data that YCHT significantly reduced liver inflammation and liver injury.

## Methods

### Chemicals and Reagents

Lithocholate (LCA), nordeoxycholate (NDCA), murideoxycholate (MDCA), hyodeoxycholate (HDCA), chenodeoxycholate (CDCA), deoxycholate (DCA), dehydrocholate (DHCA), glycocholate (GCA), taurolithocholate (TLCA), glycolithocholate (GLCA), α-muricholate (α-MCA), β-muricholate (β-MCA), ω-muricholate (ω-MCA), λ-muricholate (λ-MCA), cholate (CA), tauro λ-muricholate (Tλ-MCA), glycol λ-muricholate (Gλ-MCA), 7-dehydrocholate (7-DCA), methyl deoxycholate (methyl DCA), 6,7-diketodeoxycholate (6,7-DKDCA), tauro α-muricholate (T α-MCA), tauro β-muricholate (T β-MCA), taurocholate (TCA), tauroursodeoxycholate (TUDCA), taurohyodeoxycholate (THDCA), taurochenodeoxycholate (TCDCA), taurodeoxycholate (TDCA), glycoursodeoxycholate (GUDCA), glycohyodeoxycholate (GHDCA), glycochenodeoxycholate (GCDCA), glycodeoxycholate (GDCA), cholate-d4 (5β-Cholanic acid-3α, 7α, 12α-triol-2,2,4,4-d4, CA-d4), glycocholate-d4 (5β-Cholanic acid-3α, 7α, 12α-triol N-(Carboxymethyl)-amide-2,2,4,4-d4, GCA-d4), lithocholate-d4 (5β-Cholanic acid-3α-ol-2,2,4,4-d4, LCA-d4), and deoxycholate-d4 (5β-Cholanic acid-3α, 12α-diol-2,2,4,4-d4, DCA-d4) were purchased from Steraloids Inc. (Newport, RI). HPLC-grade methanol, acetonitrile, water, ammonium acetate, acetic acid, and α-naphthylisothiocyanate (ANIT) were obtained from Sigma-aldrich (St. Louis, MO, USA). Pure olive oil was purchased from Shanghai National Chemicals Co., Ltd. (Shanghai, China).

The kits for determination of serum alanine aminotransferase (ALT), aspartate aminotransferase (AST) and alkaline phosphatase (ALP) activity, and the levels of albumin (ALB), total bilirubin (TBIL), direct bilirubin (DBIL), triglyceride (TG), total cholesterol (TC), creatinine (Cr), and blood urea nitrogen (BUN) were purchased from Shanghai Huachen biological reagent Co. Ltd. (Shanghai, China). γ-glutamyl transpeptidase (γ-GT) assay kit was purchased from Nanjing Jiancheng Bioengineering Institute (Nanjing, China). Standard solution of hydroxyproline (HYP) was from Tokyo Chemical Industry Co., Ltd. (Tokyo, Japan). Ultrapure RNA kit was purchased from Beijing ComWin Biotech Co. Ltd (Beijing, China). RevertAid first Strand cDNA Synthesis Kit was purchased from Thermo Fisher Scientific Inc. (Vilnius, Lithuania). SYBR Green Real-Time PCR Kit was purchased from TaKaRa Biotechnology (Dalian) Co. Ltd. (Dalian, China).

### Phytochemical Analysis of YCHT

YCHT consists of *Artemisia annua* L. (Shanxi, China), *Gardenia jasminoides* Ellis (Jiangxi, China), and *Rheum Palmatum* L. (Gansu, China) in a 4.5: 1: 1.5 ratio. The medicinal herbs were purchased from Shanghai Huayu Chinese Herbs Co., Ltd. (Shanghai, China) and extracted according to our previous published patent^60^. *Artemisia annua* L. (2250 g) was first boiled in 600 L of water for 1 h, and then *Gardenia jasminoides* Ellis (500 g) and *Rheum Palmatum* L. (750 g) were added in together. Finally, the filtered solutions were concentrated into the aqueous extracts containing 0.48 g/mL raw herbs. Then the YCHT extract was dissolved in methanol and filtered through a membrane syringe filter (0.2 µm) (Millipore Co., Bedford, MA, USA) and subject to HPLC analysis. An Agilent 1200 HPLC system (Santa Clara, CA, USA) coupled with a binary pump, an automatic injector, and a diode array detector were used in this study. The separation was carried out on a Phenomenex Luna C18 (250 × 4.6 mm I.D., 5 µm) (Phenomenex, Torrance, CA, USA). The column was eluted with mobile phase (A) 0.1% acetic acid in water and (B) methanol using a linear gradient of 5–62% B over 0–75 min, 62–73% B over 75–80 min, 73–100% B over 80–85 min, held at 100% B for 10 min, then equilibration at 5% B. The flow rate was 1 mL/min and the injection volume was 20 µL. All the samples were kept at room temperature during the analysis. The detection wavelength was set at 260 nm. Agilent ChemStation software was used for peak identification and integration and the content of the constituents was calculated using standard curves of gallic acid, chlorogenic acid, geniposide, and crocin.

### Animal Models and YCHT Treatment

Male Wistar rats (160–180 g) were obtained from Vital River Laboratories (Beijing, China). The animal study was conducted in accordance with Chinese national legislation and local guidelines, and performed at Shanghai University of Traditional Chinese Medicine (Shanghai, China). The experimental protocol was approved by the Experiment Animal Center of Shanghai University of Traditional Chinese Medicine, Shanghai, China. The animals were housed, five per cage with controlled temperature (23–24 °C) and humidity (60 ± 10%) with 12 h/12 h light-dark cycles. All experimental rats were fed with standard rat chow and received water *ad libitum* throughout the experiment except those rats will be fasted for 2 h after ANIT intervention. After one week of acclimatization, the rats were randomly divided into 3 groups: the normal control group (n = 8), in which a daily dose of 10 mL/kg of body weight distilled water was given via oral gavage once a day for 4 weeks from week 9 to week 12; an ANIT treated group (model group, n = 22), in which a daily dose of 60 mg/kg of body weight ANIT (dissolved in olive oil) was given in a 0.5 mL aliquot vial oral gavage every other day for 12 weeks; YCHT treated group (n = 10), oral garage ANIT as processed in ANIT group and received a daily dose of 695 mg/kg of body weight YCHT was given in a 10 mL/kg of body weight via oral gavage once a day for 4 consecutive weeks from week 9 to week 12. The dosage of YCHT used in rat is determined according to an established formula for human−rat drug conversion^61^. At the end of 8^th^ week, 10 rats in ANIT treated group were sacrificed to judge liver injury. At the end of week 12, all animals (n = 30) were sacrificed. Serum samples were collected and stored at -80 °C pending biochemical and BAs analysis and tissue samples were collected for HYP and BAs measurement and histopathological examination.

### Biochemical analysis

Blood samples were drawn from the inferior vena cava. Serum was obtained by centrifuging the blood at 8,000 x g for 15 minutes at 4 °C. Serum ALT, AST and ALP activity, and the levels of ALB, TBIL, DBIL, TG, TC, Cr, and BUN were measured using TBA-40FR Fully Automatic Biochemical Analyzer (Toshiba, Japan). Liver γ-GT activity was detected using the colorimetry. Liver HYP content was assayed according to the previous reported method^62^.

### Histopathological evaluation, immunofluorescence analysis and qRT-PCR analysis

Liver tissues were fixed in 10% formalin, and processed for paraffin embedding. Sections (5 µm) of each sample were cut and Haematoxylin Eosin (H&E) and sirius red stained for histopathological analysis.

For immunofluorescence analysis, liver tissue slides were stained with antibodies for CK19 (10712–1-Ap, Proteintech, USA), and Collagen I (Abcam, USA), followed by staining with Goat Anti-Rabbit IgG H&L (Cy3 ®) preadsorbed (1:3000, ab6939) (Abcam, Cambridge, UK). The slides were mounted with DAPI/Antifade solution (S7113, Chemicon, USA). Positive cells were quantified using Image-Pro Plus software (Media Cybernetics, MD, USA) and detected by OLYMPUS DP71 microscopy (OLYMPUS, Japan).

Total RNA was isolated from liver tissue using TRIzol reagent (Invitrogen, Shanghai, China) according to the manufacturer’s protocol. RNA concentration was determined by a GE Nanovue Ultramicro spectrophotometer (Healthcare Bio-Sciences AB, USA). First-Strand cDNA was synthesized by reverse transcribing 1 μg of total RNA in a final reaction volume of 20 μL by the RevertAid first Strand cDNA Synthesis Kit (Thermo Fisher Scientific Inc., Lithuania). Using SYBR Green PCR kit, expression of target mRNA was measured in triplicate by the comparative cycle threshold method on the ViiA™ 7 Real-Time PCR System (Invitrogen, Life Technologies,). The forward and reverse primers for real-time PCR were designed and synthesized by Sangon Biotech (Shanghai) Co. Ltd. (Shanghai, China), and sequences are shown in Table 2. Target gene expression was normalized to β-acitin levels and presented as fold changes relative to control values (which were set at 1). For quantification of gene expression, the threshold cycle (Ct) values of all target genes were normalized to the expression of the endogenous reference (β-actin). ΔCt = Ct (target gene) - Ct (β-actin). The primers used for amplifications were listed in Table 2

**Table 2:** Primer sets for quantitative RT-PCR analysis.

Gene	Full name	ID	Sequences (Forward/Reverse 5′-3′)	Amplicon Size (bp)
IL-10	Interleukin-10	25325	AGTGGAGCAGGTGAAGAATGA-F	21
			CACGTAGGCTTCTATGCAGTTG-R	22
IL-6	Interleukin-6	24498	AGTTGCCTTCTTGGGACTGA-F	20
			ACTGGTCTGTTGTGGGTGGT-R	20
TGF-β1	Transforming growth factor β1	59086	ATTCCTGGCGTTACCTTGG-F	19
			AGCCCTGTATTCCGTCTCCT-R	20
IL-17A	Interleukin-17 A	301289	ACAAGCTCATCCCGTACCAG-F	20
			AGCCTCCAGGTTCAGTAGCA-R	20
IL-17F	Interleukin-17 F	301291	CGGATGGAGTAGAAGCAGGA-F	20
			CAGCAGCAGAGACTTGACCA-R	20
α-SMA	α-smooth muscle actin	81633	AGGGAGTGATGGTTGGAATG-F	20
			GGTGATGATGCCGTGTTCTA-R	20
FXR	Farnesoid X receptor	60351	AAGTGACCTCCACGACCAAG-F	20
			TGGCATTCTCTGTTTGCTGT-R	20
ACTB	β-actin	81822	TGACGAGGCCCAGAGCAAGA-F	20
			ATGGGCACAGTGTGGGTGAC-R	20
CAR	Constitutive androstane receptor	65035	GCATTGGATTGGAAAGGGTAA-F	21
			ACT GAT GAC CGC ACG AAG A-R	19
VDR	Vitamin D receptor	24873	GGATTCTGATGACCCGTCTG-F	20
			CGATGACCTTTTGGATGCTG-R	22
TGR5	The G-protein-coupled bile acid receptor	388443	AAGGTGGCTACAAGTGCT TC-F	20
			ATTGGCTACTGGAGTGGTAGG-R	21
NTCP	Na^+^/taurocholate Cotransporting Polypeptide	24777	CCACTTTTCTTCTTTCCTCTCCTCT-F	25
			GGTCCTTTGGAGGCTTGATTT-R	21
ASBT	Apical sodium-dependent bile acid transporter	29500	GGTACAGGTGCCGAACAGTAG-F	21
			AGATGAGTGGGAAGGTGAACA-R	21
BSEP	Bile salt export pump	83569	TGGTTTCAAGGCAATGTTAGG-F	21
			TGGGAAGCATCTGTAGCAAG-R	20
OATP1a1	Organic anion transporter	50572	AGGTTATCCATAAGGCCGTCAAAG-F	24
			TTAAGGTTAAGGGTGAAGGCCACTA-R	25
MRP2	Multi-drug related protein-2	25303	CTGGAGTTGGCTCACCTCA-F	19
			CACCCTCTGTCACTTCGGATA-R	21
MRP3	Multi-drug related protein-3	140668	GCAGTGATCGGGAGAAACA-F	19
			AAGGCATAGGACACGGAAAGA-R	21
MRP4	Multi-drug related protein-4	170924	CCTTGCGAGGGCAATTCTG-F	19
			CTTGTCGCTGTCAATGATGGTG-R	22

.

### Western blot

Liver and ileum tissue lysates were obtained, and protein concentrations were determined using a BCA protein assay kit (23225#, Thermo, California, CA). Western blot analysis was processed as previously described^63^. Equal amounts of protein samples were separated by SDS-PAGE and transferred to PVDF membranes which were blocked at room temperature for 1 h in blocking solution. The membranes were then incubated with rabbit polyclonal antibodies against BSEP, FXR, and ASBT overnight at 4 °C, followed by a 1 h incubation at room temperature with HRP-conjugated goat anti-rabbit secondary antibody. The obtained bands were then scanned and analyzed with the Odyssey quantitative western blot near-infrared system and band density was analyzed using Image J software. GAPDH and β-actin served as an internal control and experiments were performed in triplicate. All antibodies were purchased from Abcam.

### BA quantitation in serum and liver

Serum and liver BAs were measured according to the previously reported method with some modifications^64, 65^.

For serum, 50 µL serum samples were mixed with 150 μL of methanol containing internal standards (IS, 0.10 µM of CA-D4, DCA-D4, GCA-D4, and LCA-D4), and then incubated for 10 min at room temperature. After centrifugation at 20,000 g for 10 min, the supernatant was transferred to a clean tube, vacuum dried and reconstituted with 40 μL acetonitrile (with 0.1% formic acid) and 40 μL water (with 0.1% formic acid). After centrifugation, the supernatant was used for UPLC-TQMS measurement.

For liver samples, liver tissue was precisely weighed (~50 mg) and homogenized in 75 μl ice-cold 50% methanol, and then centrifuged at 20,000 g for 10 min. The supernatant was transferred to a clean tube. Then 80 μL ice-cold methanol: chloroform (3:1, v/v) was added to the remaining pellet and homogenized again. After centrifugation, the two supernatants were combined, spiked with 10 μL IS and vacuum dried. The extracts were reconstituted with 40 μL acetonitrile (with 0.1% formic acid) and 40 μL water (with 0.1% formic acid). After centrifugation, 5 μL supernatants were injected for UPLC-TQMS measurement.

The detection was performed with a Waters ACQUITY Ultra performance liquid chromatography (BEH C18 1.7 μm 2.1 × 100 mm column) coupled with Waters Xevo TQ-S triple quadrupole mass spectrometry. Data acquisition and BA quantification were performed using the TargetLynx applications manager version 4.1 (Waters Corp., Milford, MA).

### Data analysis

One-way analysis of variance and the least significant difference (LSD) test were used to investigate differences between the groups, and *P* < 0.05 was considered statistically significant. Pearson correlation analysis between BAs and bilirubin were performed with SPSS (IBM SPSS Statistics 22, USA).

## Electronic supplementary material

Supplementary Tables 1 and 2 and Supplementary Figures 1 and 2

## References

[1] Popper HC (1968). Annual review of medicine.

[2] Hofmann AF (1976). The enterohepatic circulation of bile acids in man. Advances in internal medicine.

[3] Watanabe M (2006). Bile acids induce energy expenditure by promoting intracellular thyroid hormone activation. Nature.

[4] Greim H (1972). Mechanism of cholestasis. 6. Bile acids in human livers with or without biliary obstruction. Gastroenterology.

[5] Jin F (2013). Anti-inflammatory and anti-oxidative effects of corilagin in a rat model of acute cholestasis. BMC gastroenterology.

[6] Kullak-Ublick GA, Meier PJ (2000). Mechanisms of cholestasis. Clinics in liver disease.

[7] Roma MG, Crocenzi FA, Sanchez Pozzi EA (2008). Hepatocellular transport in acquired cholestasis: new insights into functional, regulatory and therapeutic aspects. Clinical science.

[8] Guicciardi ME, Gores GJ (2005). Apoptosis: a mechanism of acute and chronic liver injury. Gut.

[9] Maher JJ, Friedman SL (1993). Parenchymal and nonparenchymal cell interactions in the liver. Seminars in liver disease.

[10] Sokol RJ, Winklhofer-Roob BM, Devereaux MW, McKim JM (1995). Generation of hydroperoxides in isolated rat hepatocytes and hepatic mitochondria exposed to hydrophobic bile acids. Gastroenterology.

[11] Sokol RJ (2001). Role of oxidant stress in the permeability transition induced in rat hepatic mitochondria by hydrophobic bile acids. Pediatric research.

[12] Fuentes-Broto L (2009). Lipid and protein oxidation in hepatic homogenates and cell membranes exposed to bile acids. Free radical research.

[13] Moran M (2009). Effect of erythropoietin on oxidative stress and liver injury in experimental obstructive jaundice. *European surgical research*. Europaische chirurgische Forschung. Recherches chirurgicales europeennes.

[14] Zhang A (2011). An *in vivo* analysis of the therapeutic and synergistic properties of Chinese medicinal formula Yin-Chen-Hao-Tang based on its active constituents. Fitoterapia.

[15] Wang X (2011). Pharmacokinetics screening for multi-components absorbed in the rat plasma after oral administration traditional Chinese medicine formula Yin-Chen-Hao-Tang by ultra performance liquid chromatography-electrospray ionization/quadrupole-time-of-flight mass spectrometry combined with pattern recognition methods. The Analyst.

[16] Xu J, Lee G, Wang H, Vierling JM, Maher JJ (2004). Limited role for CXC chemokines in the pathogenesis of alpha-naphthylisothiocyanate-induced liver injury. American journal of physiology. Gastrointestinal and liver physiology.

[17] Arab JP (2009). Effects of Japanese herbal medicine Inchin-ko-to on endotoxin-induced cholestasis in the rat. Ann Hepatol.

[18] Asakawa T (2012). The herbal medicine Inchinko-to reduces hepatic fibrosis in cholestatic rats. Pediatric surgery international.

[19] Wang X (2008). Analysis of the constituents in the rat plasma after oral administration of Yin Chen Hao Tang by UPLC/Q-TOF-MS/MS. J Pharm Biomed Anal.

[20] Wang X (2008). Metabolic urinary profiling of alcohol hepatotoxicity and intervention effects of Yin Chen Hao Tang in rats using ultra-performance liquid chromatography/electrospray ionization quadruple time-of-flight mass spectrometry. Journal of pharmaceutical and biomedical analysis.

[21] Zhou JY (1997). One case of gastritis as a result of taking Yin Chen Hao Tang. China Journal of Chinese Medicine.

[22] Liu YH, Li J, Lin MT, Zhou HH, Chen SD (2012). Modern Research and Development of Geniposide in Gardenia. Chinese Pharmaceutical Journal.

[23] Stosiek P, Kasper M, Karsten U (1990). Expression of cytokeratin 19 during human liver organogenesis. Liver.

[24] Swann JR (2011). Systemic gut microbial modulation of bile acid metabolism in host tissue compartments. Proceedings of the National Academy of Sciences of the United States of America.

[25] Dietrich CG, Ottenhoff R, de Waart DR, Oude Elferink RP (2001). Role of MRP2 and GSH in intrahepatic cycling of toxins. Toxicology.

[26] Jean PA, Roth RA (1995). Naphthylisothiocyanate disposition in bile and its relationship to liver glutathione and toxicity. Biochemical pharmacology.

[27] Hill DA, Jean PA, Roth RA (1999). Bile duct epithelial cells exposed to alpha-naphthylisothiocyanate produce a factor that causes neutrophil-dependent hepatocellular injury *in vitro*. Toxicological sciences: an official journal of the Society of Toxicology.

[28] Sullivan BP, Weinreb PH, Violette SM, Luyendyk JP (2010). The coagulation system contributes to alphaVbeta6 integrin expression and liver fibrosis induced by cholestasis. The American journal of pathology.

[29] Niemela O (2007). Biomarkers in alcoholism. Clinica chimica acta; international journal of clinical chemistry.

[30] Okada K (2007). Inchinkoto, a herbal medicine, and its ingredients dually exert Mrp2/MRP2-mediated choleresis and Nrf2-mediated antioxidative action in rat livers. American journal of physiology. Gastrointestinal and liver physiology.

[31] Wang B (2015). Yin-Chen-Hao-Tang alleviates biliary obstructive cirrhosis in rats by inhibiting biliary epithelial cell proliferation and activation. Pharmacogn Mag.

[32] Liu C (2008). Inhibition of hepatic stellate cell activation following Yinchenhao decoction administration to dimethylnitrosamine-treated rats. Hepatol Res.

[33] Liu C (2008). Effects of Yinchenhao Tang and related decoctions on DMN-induced cirrhosis/fibrosis in rats. Chin Med.

[34] Takahashi Y (2014). Japanese herbal medicines shosaikoto, inchinkoto, and juzentaihoto inhibit high-fat diet-induced nonalcoholic steatohepatitis in db/db mice. Pathol Int.

[35] Yerushalmi B, Dahl R, Devereaux MW, Gumpricht E, Sokol RJ (2001). Bile acid-induced rat hepatocyte apoptosis is inhibited by antioxidants and blockers of the mitochondrial permeability transition. Hepatology.

[36] Sodeman T, Bronk SF, Roberts PJ, Miyoshi H, Gores GJ (2000). Bile salts mediate hepatocyte apoptosis by increasing cell surface trafficking of Fas. American journal of physiology. Gastrointestinal and liver physiology.

[37] Miyoshi H, Rust C, Roberts PJ, Burgart LJ, Gores GJ (1999). Hepatocyte apoptosis after bile duct ligation in the mouse involves Fas. Gastroenterology.

[38] Patel T, Roberts LR, Jones BA, Gores GJ (1998). Dysregulation of apoptosis as a mechanism of liver disease: an overview. Seminars in liver disease.

[39] Rodrigues CM, Fan G, Ma X, Kren BT, Steer CJ (1998). A novel role for ursodeoxycholic acid in inhibiting apoptosis by modulating mitochondrial membrane perturbation. The Journal of clinical investigation.

[40] Strautnieks SS (1998). A gene encoding a liver-specific ABC transporter is mutated in progressive familial intrahepatic cholestasis. Nature genetics.

[41] Dupont J, Oh SY, O’Deen LA, Geller S (1974). Cholanoic (bile) acids in hepatic and nonhepatic tissues of miniature swine. Lipids.

[42] Queipo-Ortuno MI (2012). Influence of red wine polyphenols and ethanol on the gut microbiota ecology and biochemical biomarkers. The American journal of clinical nutrition.

[43] Mutlu EA (2012). Colonic microbiome is altered in alcoholism. American journal of physiology. Gastrointestinal and liver physiology.

[44] Chiang JY (2002). Bile acid regulation of gene expression: roles of nuclear hormone receptors. Endocrine reviews.

[45] Chiang JY (2004). Regulation of bile acid synthesis: pathways, nuclear receptors, and mechanisms. Journal of hepatology.

[46] Lefebvre P, Cariou B, Lien F, Kuipers F, Staels B (2009). Role of bile acids and bile acid receptors in metabolic regulation. Physiological reviews.

[47] Yang Y (2007). Metabonomic studies of human hepatocellular carcinoma using high-resolution magic-angle spinning 1H NMR spectroscopy in conjunction with multivariate data analysis. Journal of proteome research.

[48] Huff MW (2002). Inhibition of the apical sodium-dependent bile acid transporter reduces LDL cholesterol and apoB by enhanced plasma clearance of LDL apoB. Arteriosclerosis, thrombosis, and vascular biology.

[49] Kouzuki H, Suzuki H, Sugiyama Y (2000). Pharmacokinetic study of the hepatobiliary transport of indomethacin. Pharm Res.

[50] Ma Y (2013). Imbalanced frequencies of Th17 and Treg cells in acute coronary syndromes are mediated by IL-6-STAT3 signaling. PloS one.

[51] Xu L, Kitani A, Fuss I, Strober W (2007). Cutting edge: regulatory T cells induce CD4+ CD25-Foxp3- T cells or are self-induced to become Th17 cells in the absence of exogenous TGF-beta. Journal of immunology.

[52] Trinchieri G (2007). Interleukin-10 production by effector T cells: Th1 cells show self control. The Journal of experimental medicine.

[53] Zhang M, Xu S, Han Y, Cao X (2011). Apoptotic cells attenuate fulminant hepatitis by priming Kupffer cells to produce interleukin-10 through membrane-bound TGF-beta. Hepatology.

[54] Nelson DR (2003). Long-term interleukin 10 therapy in chronic hepatitis C patients has a proviral and anti-inflammatory effect. Hepatology.

[55] Thompson KC (1998). Primary rat and mouse hepatic stellate cells express the macrophage inhibitor cytokine interleukin-10 during the course of activation *In vitro*. Hepatology.

[56] Zandieh A (2011). Gadolinium chloride, a Kupffer cell inhibitor, attenuates hepatic injury in a rat model of chronic cholestasis. Human & experimental toxicology.

[57] Yumoto E (2002). Serum gamma-interferon-inducing factor (IL-18) and IL-10 levels in patients with acute hepatitis and fulminant hepatic failure. Journal of gastroenterology and hepatology.

[58] Ichikawa M (2010). Selectively high levels of serum interleukin 17 in a newborn infant with progressive severe cholestasis. Pediatrics.

[59] Summerfield JA, Kirk AP, Chitranukroh A, Billing BH (1981). A distinctive pattern of serum bile acid and bilirubin concentrations in benign recurrent intrahepatic cholestasis. Hepato-gastroenterology.

[60] Luo, G. A. *et al*. Composition of a traditional Chinese medicne and its application. China patent ZL201010501560.7 (2013).

[61] Chen, Q. *Experimental Methodology of Pharmacological Research in Traditional Chinese Medicine* (People’s Health Publishing House, 1993).

[62] Jamall IS, Finelli VN, Que Hee SS (1981). A simple method to determine nanogram levels of 4-hydroxyproline in biological tissues. Analytical biochemistry.

[63] Zhou Y (2016). Synergistic anti-liver fibrosis actions of total astragalus saponins and glycyrrhizic acid via TGF-beta1/Smads signaling pathway modulation. Journal of ethnopharmacology.

[64] Xie G (2013). Alteration of bile acid metabolism in the rat induced by chronic ethanol consumption. FASEB journal: official publication of the Federation of American Societies for Experimental Biology.

[65] Xie G (2015). Profiling of serum bile acids in a healthy Chinese population using UPLC-MS/MS. Journal of proteome research.

