# Supplementary material for: Herbal medicine Yinchenhaotang protects against α-naphthylisothiocyanate-induced cholestasis in rats
Source: Sci Rep. 2017 Jun 23;7:4211. doi: 10.1038/s41598-017-04536-5 (PMC5482856; doi:10.1038/s41598-017-04536-5)
Supplement: Supplementary file 1 — Supplementary Tables 1 and 2 and Supplementary Figures 1 and 2 [file 41598_2017_4536_MOESM1_ESM.pdf]

Supporting Information

Herbal medicine Yinchenhaotang protects against α-naphthylisothiocyanate-induced cholestasis in rats

Jingyu Yan, Guoxiang Xie, Chungeng Liang, Yiyang Hu, Aihua Zhao, Fengjie Huang, Ping Hu, Ping Liu, Wei Jia, Xiaoning Wang

Supplementary Table S1. Concentration of serum bile acids in rats from normal, ANIT-induced liver cholestasis model and ANIT-induced cholestasis rats treated with Yinchenhaotang from 9th week to 12th week. (μg/ml)

	Normal	8w Model	12w Model	Yinchenhaotang-treated
Primary bile acids				
CA	2.685 ± 0.865	5.746 ± 1.281	8.048 ± 1.692*	6.565 ± 1.696
GCA	0.385 ± 0.102	1.866 ± 0.686	3.226 ± 0.935*	1.187 ± 0.508
TCA	1.522 ± 0.903	2.857 ± 1.190	7.537 ± 1.839**##	1.104 ± 0.437††
CDCA	0.152 ± 0.068	0.571 ± 0.131	1.240 ± 0.296**#	0.682 ± 0.145†
GCDCA	0.013 ± 0.003	0.193 ± 0.111	0.531 ± 0.183**	0.042 ± 0.017†‡
TCDCA	0.088 ± 0.045	0.203 ± 0.085	0.575 ± 0.181**#	0.065 ± 0.014††
αMCA	0.191 ± 0.072	0.730 ± 0.184	1.176 ± 0.386	0.771 ± 0.250
TαMCA	0.242 ± 0.112	0.542 ± 0.128	1.400 ± 0.340**#	0.255 ± 0.049††

βMCA	0.289 ± 0.088	0.476 ± 0.114	0.861 ± 0.196	0.569 ± 0.125
TβMCA	0.112 ± 0.072	0.264 ± 0.095	0.951 ± 0.370*#	0.093 ± 0.031†
Secondary bile acids				
UDCA	0.196 ± 0.154	0.256 ± 0.097	0.441 ± 0.157	0.295 ± 0.089
GUDCA	0.001 ± 0.001	0.027 ± 0.013	0.067 ± 0.019	0.008 ± 0.003
TUDCA	0.021 ± 0.016	0.053 ± 0.024	0.127 ± 0.037	0.015 ± 0.007
DCA	0.378 ± 0.092	0.244 ± 0.047	0.402 ± 0.093	0.526 ± 0.137
GDCA	0.105 ± 0.035	0.139 ± 0.030	0.369 ± 0.075*	0.167 ± 0.071‡
TDCA	0.127 ± 0.026	0.119 ± 0.021	0.351 ± 0.092*##	0.119 ± 0.050†
LCA	0.001 ± 0.000	0.001 ± 0.000	0.002 ± 0.000	0.006 ± 0.002
GLCA	0.0001 ± 0.0001	0.0005 ± 0.0004	0.0013 ± 0.0007	0.0000 ± 0.0000
TLCA	0.0049 ± 0.0003	0.0059 ± 0.0009	0.0070 ± 0.0011	0.0053 ± 0.0015
ωMCA	0.426 ± 0.220	0.863 ± 0.418	1.423 ± 0.326	1.431 ± 0.438
TωMCA	0.025 ± 0.006	0.051 ± 0.012	0.140 ± 0.029**#	0.040 ± 0.016†
HCA	0.017 ± 0.005	0.055 ± 0.014	0.092 ± 0.024	0.079 ± 0.027
GHCA	0.000 ± 0.000	0.006 ± 0.003	0.021 ± 0.004**##	0.003 ± 0.002††
THCA	0.003 ± 0.002	0.009 ± 0.003	0.021 ± 0.004**##	0.002 ± 0.001††
HDCA	1.081 ± 0.226	0.960 ± 0.167	1.275 ± 0.275	2.647 ± 0.824
GHDCA	0.210 ± 0.100	0.206 ± 0.067	0.315 ± 0.080	0.194 ± 0.082
THDCA	0.370 ± 0.100	0.241 ± 0.061	0.519 ± 0.140	0.194 ± 0.066
23-norDCA	0.0014 ± 0.0013	0.0003 ± 0.0002	0.0008 ± 0.0005	0.0010 ± 0.0007

IsoDCA	0.0013 ± 0.0005	0.0022 ± 0.0007	0.0016 ± 0.0005	0.0032 ± 0.0007
6-KLCA	0.060 ± 0.010	0.067 ± 0.009	0.094 ± 0.020	0.183 ± 0.053
7-KLCA	0.004 ± 0.001	0.047 ± 0.015	0.113 ± 0.038**	0.067 ± 0.023
12-KLCA	0.101 ± 0.027	0.066 ± 0.017	0.128 ± 0.022	0.166 ± 0.040
7,12-KLCA	0.019 ± 0.004	0.058 ± 0.012	0.076 ± 0.020*	0.048 ± 0.014
isoLCA	0.002 ± 0.001	0.002 ± 0.000	0.003 ± 0.000	0.003 ± 0.001
3-KCA	0.027 ± 0.008	0.079 ± 0.015	0.118 ± 0.029**	0.122 ± 0.033
6,7-KCA	0.001 ± 0.001	0.003 ± 0.001	0.003 ± 0.001	0.007 ± 0.002
7-KDCA	0.156 ± 0.077	2.317 ± 0.681	3.432 ± 1.015*	3.040 ± 1.062
12-KCDCA	0.079 ± 0.022	0.500 ± 0.138	1.020 ± 0.271**	0.660 ± 0.191
MCA	0.039 ± 0.013	0.038 ± 0.009	0.043 ± 0.010	0.093 ± 0.028
apoCA	0.034 ± 0.014	0.024 ± 0.005	0.033 ± 0.007	0.044 ± 0.011

Note: values are expressed as mean ± SEM；* $P < 0.05$, **, $P < 0.01$ *vs*. normal group；# $P < 0.05$, ##, $P < 0.01$, *vs.* 8w model group. †$P < 0.05$, ††, $P < 0.01$, *vs.* 12w model group. ‡$P < 0.05$, *vs.* UDCA-treated group.

Supplementary Table S2. Concentration of liver bile acids in rats from normal, ANIT-induced liver cholestasis model and ANIT-induced cholestasis rats treated with Yinchenhaotang from 9th week to 12th week. (ng/mg liver tissues)

	Normal	8w Model	12w Model	Yinchenhaotang-treated
Primary bile acids				
CA	2.576 ± 1.284	4.915 ± 1.408	3.315 ± 1.055	7.748 ± 2.371
GCA	16.11 ± 5.369	23.77 ± 5.33	41.56 ± 9.58**	34.82 ± 10.75
TCA	53.26 ± 9.51	85.71 ± 8.63	89.61 ± 26.79	82.06 ± 16.66
CDCA	0.050 ± 0.026	0.154 ± 0.060	0.105 ± 0.025	0.079 ± 0.020
GCDCA	0.480 ± 0.177	2.015 ± 0.511	3.875 ± 0.762**	1.511 ± 0.411†
TCDCA	2.259 ± 0.454	5.066 ± 0.932	8.685 ± 1.714**	4.617 ± 0.577††
αMCA	0.128 ± 0.055	0.473 ± 0.169	0.383 ± 0.093	0.847 ± 0.307
TαMCA	6.073 ± 0.743	17.140 ± 1.724**	23.780 ± 4.312**	15.510 ± 2.112†
βMCA	0.363 ± 0.098	0.768 ± 0.265	0.893 ± 0.163	1.602 ± 0.500
TβMCA	5.917 ± 1.259	7.921 ± 1.683	16.09 ± 4.083*#	7.868 ± 1.391†
Secondary bile acids				
UDCA	0.059 ± 0.014	0.126 ± 0.027	0.140 ± 0.022	0.155 ± 0.036
GUDCA	0.081 ± 0.026	0.526 ± 0.144	0.917 ± 0.168	0.574 ± 0.150
TUDCA	3.705 ± 1.731	2.373 ± 0.580	4.732 ± 0.855	3.151 ± 0.648
DCA	0.264 ± 0.134	0.076 ± 0.013	0.073 ± 0.015	0.154 ± 0.052
GDCA	3.192 ± 1.073	2.083 ± 0.414	3.513 ± 0.890	4.436 ± 1.549

TDCA	5.541 ± 1.816	2.793 ± 0.575	3.610 ± 0.990	6.037 ± 1.730
LCA	0.010 ± 0.005	0.044 ± 0.019	0.015 ± 0.010	0.003 ± 0.003
GLCA	0.028 ± 0.011	0.043 ± 0.007	0.046 ± 0.010	0.077 ± 0.020
TLCA	0.173 ± 0.031	0.113 ± 0.023	0.120 ± 0.035	0.258 ± 0.097
ωMCA	0.291 ± 0.123	0.638 ± 0.327	0.599 ± 0.154	1.357 ± 0.443
TωMCA	1.756 ± 0.322	2.819 ± 0.725	3.786 ± 0.644**	3.970 ± 0.645
HCA	0.010 ± 0.004	0.025 ± 0.010	0.033 ± 0.009	0.038 ± 0.014
GHCA	0.022 ± 0.010	0.106 ± 0.026	0.180 ± 0.031**	0.117 ± 0.033
THCA	0.074 ± 0.019	0.234 ± 0.024**	0.330 ± 0.045**##	0.218 ± 0.032†
HDCA	0.788 ± 0.238	0.266 ± 0.046	0.335 ± 0.074	0.952 ± 0.301
GHDCA	2.220 ± 0.922	1.739 ± 0.421	1.844 ± 0.405	2.638 ± 0.750
THDCA	3.979 ± 1.758	1.442 ± 0.734	1.407 ± 0.961	3.593 ± 1.182
IsoDCA	0.030 ± 0.005	0.024 ± 0.006	0.017 ± 0.006	0.018 ± 0.005
6-KLCA	0.033 ± 0.008	0.035 ± 0.004	0.066 ± 0.018	0.187 ± 0.059
7-KLCA	0.005 ± 0.002	0.013 ± 0.002	0.011 ± 0.003	0.010 ± 0.006
12-KLCA	0.037 ± 0.011	0.040 ± 0.011	0.053 ± 0.010	0.100 ± 0.029
7,12-KLCA	0.056 ± 0.013	0.046 ± 0.012	0.091 ± 0.018	0.064 ± 0.015
3-KCA	0.019 ± 0.011	0.023 ± 0.006	0.028 ± 0.006	0.037 ± 0.011
7-KDCA	0.207 ± 0.111	3.432 ± 1.284	2.044 ± 0.610	6.440 ± 2.176
12-KCDCA	0.013 ± 0.006	0.171 ± 0.060	0.423 ± 0.207	0.505 ± 0.199
MCA	0.138 ± 0.034	0.060 ± 0.011	0.075 ± 0.017	0.200 ± 0.062

Note: values are expressed as mean ± SEM； * $P < 0.05$, **, $P < 0.01$ *vs*. normal group；# $P < 0.05$, ##, $P < 0.01$, *vs.* 8w model group. †$P <0.05$, ††, $P <0.01$, *vs.* 12w model group.

Supplementary Fig. S1

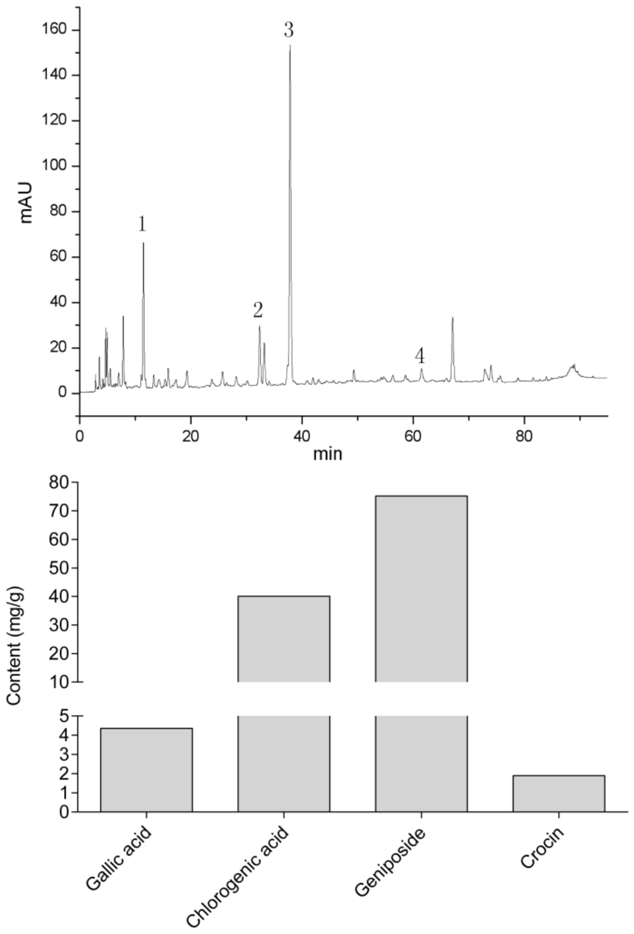

Representative HPLC chromatogram of the YCHT extraction and main compound content in the YCHT extraction.

Supplementary Fig. S2

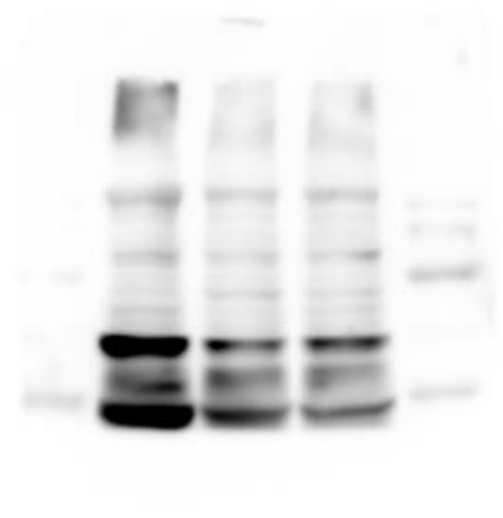

Full-length gels and blots for liver BSEP (3s).

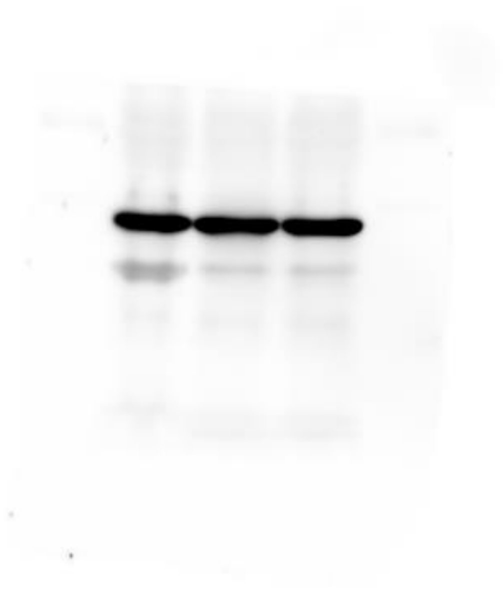

Full-length gels and blots for liver GAPGH (3s).

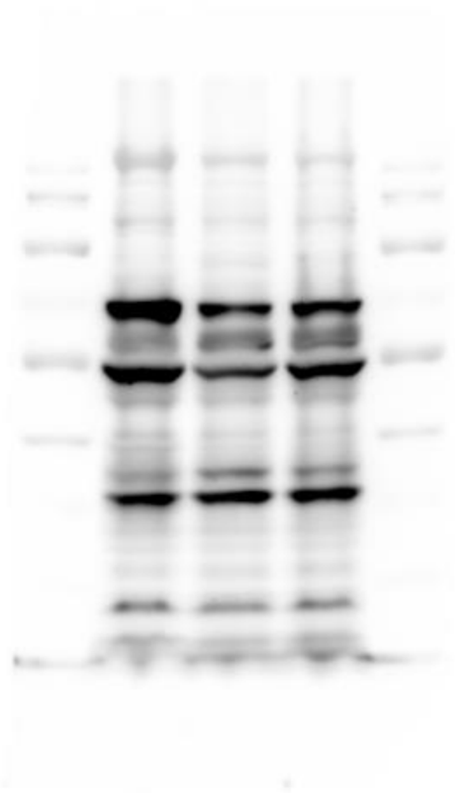

Full-length gels and blots for liver FXR+GAPDH (30s).

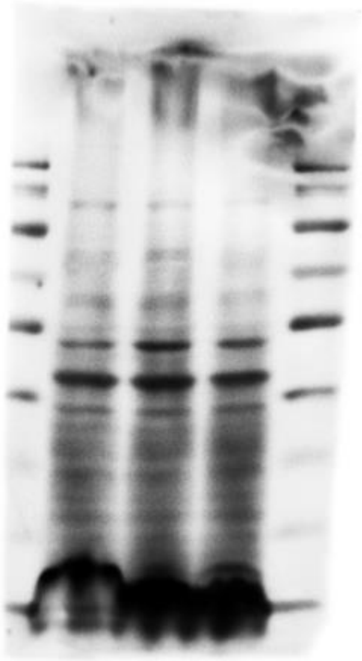

Full-length gels and blots for ileum ASBT+GAPDH (60s).
